# 3D printing visualization combined with home–school–hospital collaboration for clinical pharmacology teaching

**DOI:** 10.3389/fphar.2026.1797204

**Published:** 2026-05-26

**Authors:** Xinyu Wang, Wenwen Hou, Xiaofen Yu, Qiongting Luo, Zheng Wang

**Affiliations:** 1 Hangzhou Medical College, The Affiliated People’s Hospital of Hangzhou Medical College, Zhejiang Provincial People’s Hospital, Hangzhou, China; 2 Xiasha Campus of Hangzhou First People’s Hospital (Hangzhou Rehabilitation Hospital), Hangzhou, Zhejiang, China

**Keywords:** 3D printing visualization, clinical pharmacy education, collaborative learning, medical education innovation, pharmacology teaching

## Abstract

**Background:**

Clinical pharmacology teaching is often limited by abstract content and insufficient integration of anatomical structure, physiological regulation, and drug targets. Traditional teaching models make it difficult for interns to transform theoretical knowledge into clinical application ability. Based on embodied cognition theory, 3D-printed visualization models can provide intuitive and perceptual learning support. This study aimed to construct an innovative teaching model integrating School-Family-Physician Collaboration (SFPC) and 3D intelligent printing teaching (3DIPT) for clinical pharmacology education.

**Methods:**

A non-randomized longitudinal pre-post intervention study was conducted among clinical interns. The experimental group received integrated teaching using 3D-printed anatomical and pharmacological models combined with real family and social scenarios. The control group received traditional theoretical teaching. Teaching effects were evaluated using objective test scores, target recognition accuracy, and questionnaire-based learning initiative and satisfaction.

**Results:**

The baseline data of the two groups were balanced and comparable (all P > 0.05). After intervention, the scores of the observation group were significantly higher than those of the control group in pharmacology knowledge test (67.23 ± 10.62 vs. 63.28 ± 7.35, P = 0.0028), SMC (5.618 ± 0.106 vs. 5.574 ± 0.104, P = 0.0232), self-directed learning ability (117.85 ± 1.78 vs. 117.10 ± 1.47, P = 0.0212), doctor-patient communication ability (21.54 ± 0.76 vs. 21.23 ± 0.73, P = 0.0204), and MSTLR (62.88 ± 8.36 vs. 58.73 ± 6.35, P = 0.0315), with statistically significant differences.

**Conclusion:**

The integrated SFPC–3DIPT teaching model can significantly improve interns’ mastery of urogenital system pharmacology, enhance their understanding of physiological and pharmacological mechanisms, and increase learning initiative and clinical application ability. This model provides a feasible and effective reference for the reform of clinical pharmacology teaching.

## 1 Introduction

Clinical pharmacology teaching has long faced the pain point of “disconnection between abstract mechanisms and clinical application.”

Traditional theoretical teaching or two-dimensional courseware struggles to help interns build a complete logical chain: anatomical structure–hormonal regulation–drug targets–clinical efficacy. Taking benign prostatic hyperplasia (BPH) and menopausal endocrine disorders as examples, knowledge becomes highly abstract. BPH involves target differences among α-blockers, 5α-reductase inhibitors, and other drug classes (Eleazu et al., 2017). Menopausal disorders include complex regulation of estrogens, progestogens, and newer non-hormonal agents (Mehta et al., 2021). These points are key and difficult content in clinical medicine postgraduate examinations. However, interns often cannot master them accurately through classroom learning alone. Traditional teaching leaves students unable to link physiological processes with drug mechanisms. Menopausal hormone therapy involves complex endocrine regulation and multiple clinical risks and benefits, requiring accurate understanding of hormone pathways and drug safety profiles (Al-Rousan et al., 2018; Khalili, 2016; Kling et al., 2023). According to embodied cognition theory, concrete, perceptual, and visual learning experiences significantly improve understanding of abstract biomedical principles (Wilson, 2002). Three-dimensional (3D) printed models support embodied learning by providing spatial and intuitive representations of human anatomy and physiology (Fleming et al., 2020). This approach helps learners connect structural anatomy, physiological regulation, and pharmacological action more effectively. Existing studies report that under traditional teaching, interns’ accuracy in identifying urogenital drug targets is below 30%.Recognition of links between side effects and anatomical structure is even lower, at less than 25% (US Preventive Services Task Force et al., 2017). This gap directly reduces the accuracy and safety of clinical medication decisions. Although international and domestic guidelines clarify clinical pathways for menopausal hormone therapy, interns struggle to translate guidelines into practice. A major reason is the lack of visualization tools and real-scenario support (Bolton, 2016). Meanwhile, purely theoretical learning lacks feedback for social value recognition.

This leads to low learning interest and insufficient initiative among interns. The School-Family-Physician Collaborative (SFPC) model offers a new solution. It connects pharmacology knowledge with high-prevalence real cases among relatives and friends. Examples include BPH (prevalence >50% in men over 60) and menopausal syndrome (incidence >40% in women over 45). Interns gain social recognition when explaining medication logic to family members. This creates a virtuous cycle of “learning–application–feedback” (Cintron et al., 2017). Our research team has extensive experience in 3D Intelligent Printing Teaching (3DIPT) for clinical training. Previously developed urological anatomical models have achieved good results in practical teaching (Wang et al., 2024; Wei et al., 2019; Yang et al., 2022; Yu et al., 2022; Zhang et al., 2022). This study innovatively integrates SFPC with 3DIPT for clinical pharmacology teaching. The 3D models clearly label hormone sources, drug targets, and signal transduction pathways on prostate, ovary, and uterine structures. This design achieves integrated teaching of “pathological anatomy visualization–hormone pathway analysis–pharmacological target localization” (Passos et al., 2021). The intervention aims to improve interns’ comprehensive ability in urogenital pharmacotherapy. It also provides a new model for reforming clinical pharmacology education.

## 2 Materials and methods

### 2.1 Study design and participants

This non-randomized prospective cohort study was conducted in a provincial hospital in China from February 2025 to August 2025. A total of 132 undergraduate clinical medicine interns were enrolled. Traditional randomized controlled grouping was not used in this study. This design was chosen to better align with actual clinical internship management. It also helped avoid scheduling conflicts and poor cooperation related to rigid randomization. Grouping was determined according to real-world internship arrangements. The observation group (SFPC + 3DIPT model) included interns from non-local medical colleges. These interns lived in rented housing near the hospital and had flexible spare time. They could fully participate in SFPC discussions and 3DIPT model-related learning. The control group (traditional internship teaching) included interns from local medical colleges. These interns commuted daily between home and school and had limited extra time for additional teaching activities. Each group initially included 66 interns. Sample size estimation was performed using SPSS 23.0. Statistical descriptions included composition ratios and mean ± standard deviation. The total sample size was defined as N = n_1_ + n_2_, with a 1∶1 allocation ratio (k = 1). Based on previous studies and pilot data, the standard mean difference (σ) was set at 3.5. The between-group mean difference (ε) was 2.1, with α = 0.05 and β = 0.2. Statistical calculations showed a minimum required sample size of 60 cases per group. To account for potential attrition (e.g., incomplete questionnaires, loss to follow-up, early internship termination), the sample size was increased by approximately 10%.Finally, 66 interns were included in each group, for a total of 132 participants. After the intervention, 120 interns completed all procedures and data collection (60 in the observation group, 60 in the control group). Twelve interns were excluded due to incomplete data or early termination of the internship.

Statistical analysis was performed using the final valid sample.

### 2.2 Inclusion and exclusion criteria

#### 2.2.1 Inclusion criteria

1. Aged ≥21 years; (2) Completed undergraduate theoretical learning at school and currently in the final year of internship, entering the clinical internship stage; (3) No mental illness or psychological abnormalities; (4) Understanding of the research purpose and voluntary participation.

#### 2.2.2 Exclusion criteria

1. Interruption of internship due to special circumstances; (2) Interns who took consecutive leave for more than 3 days or cumulative leave for more than 7 days during the internship period; (3) Participation in training of other teaching methods; (4) Incomplete data.

### 2.3 Data collection methods

Multiple data collection methods were adopted in this study, including questionnaires, self-assessment reports and knowledge tests. These methods were used to comprehensively evaluate the effectiveness of the SFPC+3DIPT teaching model in the observation group. The evaluation contents covered the improvement of undergraduate clinical medicine interns' multiple abilities, including pharmacology knowledge mastery, medical curiosity, self-directed learning ability, doctor-patient communication ability and transformative learning ability. During the whole data collection process, strict measures were taken to ensure data accuracy and reliability. Meanwhile, the privacy and legitimate rights of all participants were fully protected. We also implemented standardized operation procedures to minimize potential research bias and data errors.

### 2.4 Teaching interventions

**FIGURE 1:**
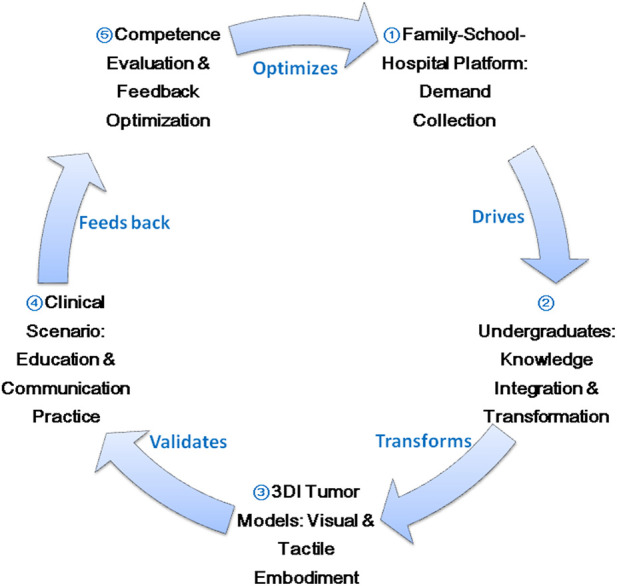
The closed-loop teaching framework of 3D Intelligent Printing (3DI) tumor models integrated with the Family-School-Hospital WeChat platform for clinical medicine undergraduates. The framework comprises five sequential phases: demand collection via the collaborative platform, knowledge integration and transformation by undergraduates, visual and tactile embodiment using 3DI tumor models, clinical scenario-based health education and communication practice, and competence evaluation with iterative feedback optimization.

- Control Group: Interns received traditional internship teaching according to the standard Clinical Internship Manual, including traditional bedside teaching, case discussions, and theoretical lectures.
- Observation Group: The observation group adopted the SFPC+3DIPT combined model based on the WeChat platform. Taking real health needs as the entry point, it built a closed-loop teaching system of “information exchange-case discussion-model assistance-knowledge internalization” through multi-subject collaboration and 3DIPT technology empowerment. The specific implementation path is as follows (Figure 1).

#### 2.4.1 Platform operation (core link of SFPC)

The intervention centered on real health needs in interns’ families. Parents were encouraged to raise consultation questions and discuss practical issues such as disease diagnosis, treatment and medication. This mechanism encouraged interns to actively conduct case analysis and targeted literature review. For typical related diseases, teachers provided guidance based on professional experience and clinical practice advantages. Interns, teachers and family members jointly reviewed anatomical and imaging literature as well as textbook contents.

Combined with 3DIPT technology, targeted pathological models were printed. This approach helped interns thoroughly understand therapeutic mechanisms, including hormone pathway analysis and pharmacological target localization. Clinical interns often have irregular schedules, including day shifts, night shifts, outpatient duties and ward work. Therefore, all platform interactions and discussions were arranged flexibly during clinical gaps. A fixed weekly online summary discussion was held to ensure teaching continuity. All family-related cases were de-identified to protect privacy. Written informed consent was obtained for the use of family health information,and all procedures were approved by the ethics committee.

#### 2.4.2 Interactive discussion

For complex organ-related knowledge and difficult cases, regular WeChat group video conferences were conducted.

Each session lasted 15–20 min. Participants included department teachers, 3DIPT professional technicians and all interns in the observation group. Discussion contents covered anatomical structure analysis, drug target identification, model printing quality inspection and key points of clinical application.

#### 2.4.3 3DIPT model preparation

The study used hospital Multi-Detector Computed Tomography (MDCT) image data.

**FIGURE 2:**
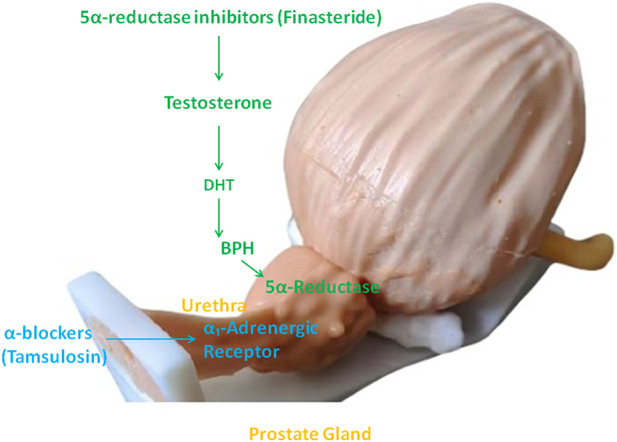
Pathophysiological mechanism of benign prostatic hyperplasia (BPH) and pharmacological targets of first-line therapeutic agents. Testosterone is converted to dihydrotestosterone (DHT) by 5α- reductase, driving prostatic hyperplasia and urethral compression. 5α-reductase inhibitors (e.g., finasteride) reduce DHT synthesis and induce prostate volume regression, while α1-adrenergic receptor blockers (e.g., tamsulosin) relax urethral smooth muscle to improve urinary flow and relieve lower urinary tract symptoms.

**FIGURE 3:**
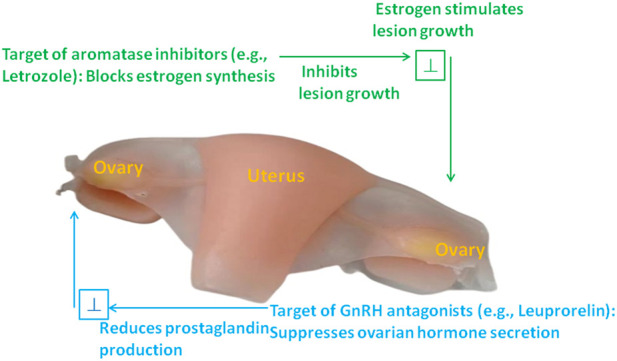
3D-printed model of the uterus and ovaries illustrating the pharmacology of endometriosis. Yellow labels indicate anatomical structures: the uterus and bilateral ovaries. Green annotations show the estrogen-driven growth of endometriotic lesions and their inhibition by aromatase inhibitors (e.g., letrozole). Blue annotations illustrate the mechanism of GnRH antagonists (e.g., leuprorelin), which suppress ovarian hormone secretion, reduce prostaglandin production, and relieve pelvic pain. (denoted by the ⊥ symbol).

Zhongnan e3D Digital Medical Virtual Software V17.06 (China) was used for three-dimensional reconstruction of target organs and lesions. Ultimaker Cura 4.4.1 open-source slicing software (USA) was used to analyze lesion tissues and related ducts. Corresponding G-code instructions were generated. Model printing was performed using Zhongrui Zhichuang SL600 stereolithography rapid prototyping equipment (China). A composite material of soft resin and hard resin was applied. The printed organ models retained complete anatomical layers and clear lesion characteristics. Professional dyeing was used to meet clinical teaching needs (Figures 2, 3). All model production and printing were arranged by experienced teachers during spare time. This schedule did not interfere with routine clinical work.

### 2.5 Outcome measures

#### 2.5.1 Primary outcome

Simulated postgraduate entrance examination medical comprehensive pharmacology knowledge test: A 100-point test was developed by the research team based on real exam questions from 2000 to 2025, reflecting interns' ability to apply theoretical knowledge to clinical practice. This test is crucial for undergraduate clinical medicine interns as it verifies their mastery of core clinical knowledge points.

#### 2.5.2 Secondary outcomes

- Social Medical Curiosity (SMC) Scale: The Chinese version of the scale translated and revised by Yang et al. (2025) includes two dimensions (Intellectual Medical Curiosity [IMC] and Social Medical Curiosity [SMC]) with a total of 10 items. The overall Cronbach’s α coefficient of the scale is 0.852, and the Cronbach’s α coefficients of each dimension are 0.796 and 0.866, respectively, indicating good reliability for evaluating medical curiosity among Chinese undergraduate medical students. Consistent with prior research showing minimal changes in IMC during the learning period (Bugaj et al., 2023), this study excluded the IMC dimension and only adopted the SMC scale for outcome assessment.
- Self-Directed Learning Ability Assessment Scale for Medical Students: Constructed by Wang et al. (2014), this scale covers four dimensions (learning goal setting, learning plan execution, learning resource utilization, and learning effect reflection) with 28 items in total. It uses a five-point Likert scale (1 = “completely inconsistent” to 5 = “completely consistent”). The overall Cronbach’s α coefficient of the scale is 0.893, and the Cronbach’s α coefficients of each dimension range from 0.782 to 0.861, showing satisfactory reliability for the quantitative evaluation of medical students' self-directed learning ability.
- Doctor-Patient Communication Ability (SEGUE Scale): The localized SEGUE framework validated by Shen and Sun (2017) was used to assess interns' doctor-patient communication ability. The scale consists of five core communication phases (Setting the stage, Eliciting information, Giving information, Understanding the patient’s perspective, Ending the encounter) with 25 items, adopting a binary “yes/no” scoring system (total score: 0–25 points). Higher scores represent stronger doctor-patient communication skills, which can effectively evaluate the impact of the SFPC+3DIPT teaching model on interns' clinical communication competence.
- Transformative Learning Readiness Scale for Medical Students (MSTLR): Developed by He et al. (2025), this scale includes four dimensions (critical thinking, knowledge transfer ability, innovation awareness, and practical application willingness) with 25 items in total. It uses a five-point Likert scale (1 = “completely not possessed” to 5 = “fully possessed”). The overall Cronbach’s α coefficient of the scale is 0.887, and the Cronbach’s α coefficients of each dimension range from 0.773 to 0.859, which can reliably assess medical students' readiness to transform theoretical knowledge into clinical practical abilities.

### 2.6 Statistical analysis

Data were collected at baseline (pre-intervention) and after 6-month intervention. All statistical analyses were performed using SPSS 29.0 (SPSS Inc., Chicago, IL, USA).

Categorical variables were presented as frequencies and percentages, and compared using the chi-square test or Fisher exact test as appropriate. Continuous variables were tested for normality using the Shapiro-Wilk test. Normally distributed data were expressed as mean ± standard deviation (SD).

Between-group comparisons at baseline were conducted using independent-samples t-test to confirm baseline comparability.

Within-group changes from pre-to post-intervention were analyzed using paired-samples t-test.

The primary intervention effect was evaluated by comparing the post-intervention scores and the pre–post change scores between groups using independent-samples t-test, which accounted for baseline differences.

Effect sizes (Cohen’s d), 95% confidence intervals (CI), and P-values were reported for all between-group comparisons. A two-sided P < 0.05 was considered statistically significant.

## 3 Ethics approval

This preliminary study was conducted in strict accordance with the principles outlined in the 2024 Declaration of Helsinki (World Medical Association, 2024 revision). Ethical approval was granted by the Institutional Review Board of Zhejiang Provincial People’s Hospital (Affiliated People’s Hospital of Hangzhou Medical College) prior to the initiation of the study, with the reference number: Zhejiang Provincial People’s Hospital Ethical Review 2025 (488).

Written informed consent was obtained from all individual participants. Prior to consenting, participants were provided with a detailed explanation of the study purpose, procedures, potential risks and benefits, and their right to withdraw from the study at any time without penalty. Voluntary submission of completed responses was explicitly defined as confirmation of consent to participate.

In addition, since this study involved family members in the school-family-hospital collaborative communication mechanism, written informed consent was also obtained from all participating family members prior to their involvement. All interactions between interns and family members were conducted in a private and confidential manner, and all health-related information shared during these interactions was anonymized and protected in accordance with the hospital’s privacy protocols to ensure the confidentiality and privacy of all participants, including family members.

To protect participant privacy and ensure data confidentiality, no personally identifiable information was collected throughout the study process. All collected data were anonymized and stored securely in a password-protected database accessible only to the research team.

## 4 Results

### 4.1 Baseline characteristics

There were no statistically significant differences in baseline characteristics (age, gender, ethnicity, only child status, GPA) between the two groups (all P > 0.05), indicating similar baseline conditions, which were suitable for comparing the intervention effects of SFPC+3DIPT (Table 1).

**TABLE 1:** Baseline characteristics of participants in the control and observation groups.

Parameters	Traditional teaching group (n = 60)	SFPC+3DPIT teaching group (n = 60)	Test statistic (t/χ^2^)	P value
Age (years)	22.43 ± 0.95	22.48 ± 0.93	0.291	0.771
Gender (M/F)	27(45.00%/43(55.00%)	28(46.67%)/42(53.33%)	0.046	0.830
Ethnicity (Han)	54 (90.00%)	53(88.33%)	0.086	0.770
Residence (Urban/Rural)	22(36.67%)/38(63.33%)	23(38.33%)/37(61.67%)	0.036	0.850
Only-child (yes)	24 (40.00%)	25 (41.67%)	0.034	0.853
Undergraduate GPA	4.91 ± 0.86	4.84 ± 0.80	0.462	0.645

Continuous variables are presented as mean ± standard deviation and compared using independent samples t-test; categorical variables are presented as n (%) and compared using Pearson’s chi-square test.

### 4.2 Comparison of outcome indicators before and after intervention

#### 4.2.1 Pharmacology knowledge test score

At baseline, pharmacology knowledge test scores were comparable between the two groups (56.00 ± 6.29 vs. 57.50 ± 5.24, t = 1.502, df = 118, P = 0.1357, 95% CI: −3.557 to 0.488).

**FIGURE 4:**
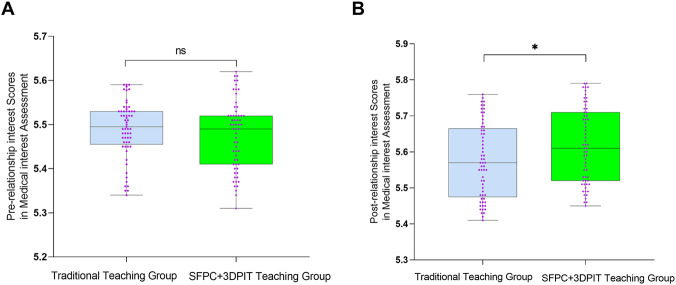
Comparison of interest scores in medical learning between the two groups before and after intervention. **(A)** Pre-intervention interest scores; **(B)** Post-intervention interest scores. Data are presented as mean ± standard deviation. P < 0.05, *P < 0.01 compared with the control group after intervention.

After intervention, the observation group achieved significantly higher scores than the control group (68.23 ± 10.62 vs. 63.28 ± 7.35, t = 3.056, df = 118, P = 0.0028, 95% CI: 1.742–8.158) (Figure 4). This difference remained significant after accounting for baseline values, indicating a greater improvement in the intervention group.

#### 4.2.2 Social Medical Curiosity (SMC) score

At baseline, the SMC scores were comparable between the two groups (5.489 ± 0.062 vs. 5.476 ± 0.081, t = 0.9369, df = 120, P = 0.3507, 95% CI: −0.0382 to 0.0137).

**FIGURE 5:**
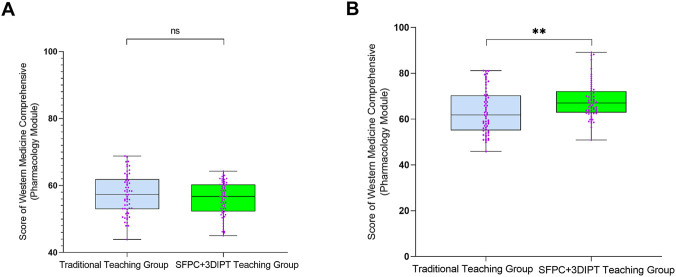
Comparison of SMC scores between the two groups before and after intervention. **(A)** Pre-intervention SMC scores; **(B)** Post-intervention SMC scores. Data are presented as mean ± standard deviation. P < 0.05, *P < 0.01 compared with the control group after intervention.

After the intervention, the observation group achieved significantly higher SMC scores than the control group (5.617 ± 0.106 vs. 5.574 ± 0.104, t = 2.299, df = 120, P = 0.0232, 95% CI: 0.0060–0.0809) (Figure 5). This difference remained significant after accounting for baseline values, indicating a greater improvement in the intervention group’s interest in the pathogenesis and treatment of diseases affecting relatives and friends.

#### 4.2.3 Self-directed learning ability score

At baseline, self-directed learning ability scores were comparable between the two groups (89.40 ± 9.00 vs. 91.81 ± 8.60, t = 1.498, df = 118, P = 0.1367, 95% CI: −5.593 to 0.7746).

**FIGURE 6:**
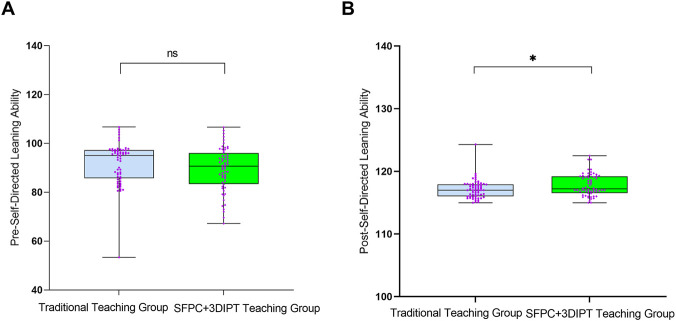
Comparison of self-directed learning ability scores between the two groups before and after intervention. **(A)** Pre-intervention self-directed learning ability scores; **(B)** Post-intervention self-directed learning ability scores. Data are presented as mean ± standard deviation. *P < 0.05 compared with the control group after intervention.

After intervention, the observation group achieved significantly higher scores than the control group (117.85 ± 1.78 vs. 117.10 ± 1.47, t = 2.335, df = 118, P = 0.0212, 95% CI: 0.1046–1.272) (Figure 6). This difference remained significant after accounting for baseline values, indicating a greater improvement in self-directed learning autonomy and initiative in the intervention group.

#### 4.2.4 Doctor-patient communication ability score

At baseline, doctor-patient communication ability scores were comparable between the two groups (17.34 ± 0.51 vs. 17.19 ± 0.49, t = 1.608, df = 118, P = 0.1104, 95% CI: −0.0339–0.3272).

**FIGURE 7:**
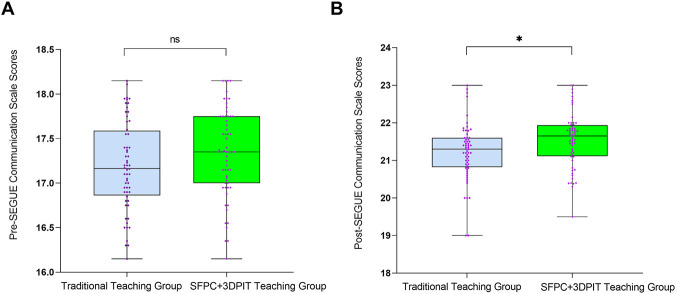
Comparison of SEGUE communication scale scores between the two groups before and after intervention. **(A)** Pre-intervention SEGUE communication scale scores; **(B)** Post-intervention SEGUE communication scale scores. Data are presented as mean ± standard deviation. *P < 0.05 compared with the control group after intervention.

After intervention, the observation group achieved significantly higher scores than the control group (21.54 ± 0.76 vs. 21.23 ± 0.73, t = 2.351, df = 119, P = 0.0204, 95% CI: 0.0502–0.5863) (Figure 7). This difference remained significant after accounting for baseline values, indicating a greater improvement in doctor-patient communication skills among interns in the intervention group, likely due to the home-school-hospital platform experience of explaining diseases and popularizing pharmacology knowledge to relatives and friends.

#### 4.2.4 Transformative Learning Readiness Scale for Medical Students (MSTLR) Score

At baseline, Learning Readiness Scale for Medical Students (MSTLR) scores were comparable between the two groups (43.46 ± 8.08 vs. 40.81 ± 7.05, t = 1.340, df = 118, P = 0.1828, 95% CI: −1.267–6.570).

**FIGURE 8:**
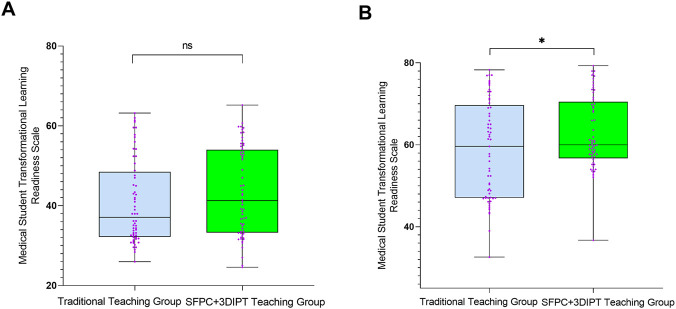
Comparison of MSTLR (Medical Student Transformational Learning Readiness) scores between the two groups before and after intervention. **(A)** Pre-intervention MSTLR scores; **(B)** Post-intervention MSTLR scores. Data are presented as mean ± standard deviation. *P < 0.05 compared with the control group after intervention.

After intervention, the observation group achieved significantly higher scores than the control group (62.88 ± 8.36 vs. 58.73 ± 6.35, t = 2.176, df = 120, P = 0.0315, 95% CI: 0.3746–7.937) (Figure 8). This difference remained significant after adjusting for baseline values, indicating a greater improvement in transformative learning ability among interns in the intervention group.

## 5 Discussion

### 5.1 Core result interpretation: Synergistic effect of SFPC+3DIPT

The results of this study showed that interns in the observation group (SFPC+3DIPT model) had significantly higher scores in pharmacology and internal medicine drug therapy knowledge in the simulated medical comprehensive examination than those in the control group. They also showed significant improvements in learning interest, self-directed learning, doctor-patient communication, and transformative learning ability. This result is highly consistent with the conclusions of existing studies.3DIPT technology can realize integrated teaching of “pathological anatomy visualization-hormone pathway analysis-pharmacological target localization” through the advantage of spatial visualization. It helps interns quickly establish the association between anatomical structures and pharmacological mechanisms, and significantly improves the understanding efficiency of complex knowledge points (Passos et al., 2021). The relative and friend case scenarios linked by SFPC can enable learners to deepen knowledge memory through emotional resonance, increasing the retention rate of drug safety-related cognition by more than 40% (Cintron et al., 2017). These findings can be further supported by embodied cognition theory. Wilson (2002) emphasized that cognitive processes rely on concrete physical experience and intuitive interaction rather than abstract memorization alone. Meanwhile, Fleming et al. (2020) confirmed in a systematic review and meta-analysis that 3D-printed models effectively improve learning outcomes in medical education. The SFPC+3DIPT model in this study conforms to the core idea of embodied learning. It turns abstract pharmacological mechanisms into visualized and experiential content, thus enhancing comprehension and application ability. Specifically, in the teaching of BPH drugs, the 3DIPT model clearly presents the relaxation mechanism of α-blockers (tamsulosin) on urethral smooth muscle and the inhibitory pathway of 5α-reductase inhibitors (finasteride) on androgen conversion. This is completely consistent with the research conclusions on drug action mechanisms published in Front Pharmacol (Eleazu et al., 2017). In the teaching of menopausal endocrine drugs, the model accurately labels estrogen and progesterone receptor targets and GnRH agonist regulatory pathways. These are highly consistent with the core guidelines for menopausal hormone therapy (Mehta et al., 2021). These visual labels not only help interns accurately grasp the differences in drug targets. They also deepen their practical understanding of the clinical indications, contraindications, and adverse reactions of drugs through the discussion of relative and friend cases linked by SFPC (Prior, 2018; Santen et al., 2017). The 3D printing-based visualization model integrated with home–school–hospital collaboration aligns well with the core tenets of embodied cognition theory. Consequently, this model significantly enhanced learners’ understanding of the underlying physiological principles, as evidenced by statistically significant improvements in pharmacology and pharmacy knowledge directly related to physiological principles, as well as improved comprehension and application of these principles in simulated clinical contexts.

### 5.2 Clinical significance of scale results: Multi-dimensional improvement of interns' comprehensive learning literacy

This study also used secondary indicators to observe the effect of SFPC+3DIPT assisted teaching.

Four scales including medical interest, self-directed learning, doctor-patient communication, and transformative learning ability were adopted.

The results confirmed that this model can achieve an all-round improvement of interns' medical literacy.

All scales are mature application tools in the field of medical education.

They provide a reliable basis for the scientificity of scale selection.

Existing studies have also shown that visualization teaching can significantly stimulate students' willingness to learn actively (Kim and Brinton, 2021).

The application of knowledge in real clinical scenarios can effectively enhance self-directed learning ability and knowledge transformation efficiency (Santen et al., 2017).

These findings echo the scale results of this study.

They further confirm the teaching effectiveness of the SFPC+3DIPT model.

### 5.3 Explanation of grouping design and statistical processing: Conforming to clinical reality and ensuring result reliability

Combined with the actual scenario of clinical internship, this study did not adopt traditional randomized controlled grouping.

Instead, grouping was performed according to interns' commuting and time availability.

Interns from non-local medical colleges and universities, who lived in rental housing near the hospital and had flexible time, were included in the observation group (SFPC+3DIPT teaching). Interns from local medical colleges and universities, who commuted between home and school and had no extra spare time to participate, were included in the control group (traditional internship teaching).

This grouping method fully conforms to the reality of clinical internship management.

It avoids internship arrangement conflicts and poor student cooperation caused by random grouping, and improves the operability of the study.

At the statistical level, this study performed balance tests on the baseline data of interns in the two groups (age, gender, ethnicity, only child status, place of residence, school baseline GPA, etc.) using SPSS software.

The results showed that there were no statistically significant differences in the baseline data between the two groups (P > 0.05).

Although baseline indicators were well balanced between the two groups, residual confounding factors and selection bias related to non-random allocation could not be completely excluded.

Baseline balancing analysis reduced the influence of measurable confounding factors to a certain extent, ensuring the basic comparability and credibility of the research results.

### 5.4 Extended value of the Team’s 3DIPT achievements: Cross-disciplinary breakthrough from clinical practice to basic teaching

The research team has previously achieved a series of results in the field of 3DIPT clinical training models.

The constructed urological anatomical models and other models have been widely used in the training of clinical physicians and standardized training students with remarkable results (Wang et al., 2024; Wei et al., 2019; Yang et al., 2022; Yu et al., 2022; Zhang et al., 2022). On this basis, this study further expands the pharmacological visualization function of 3DIPT models. Combined with the SFPC collaborative model, it realizes a cross-disciplinary leap from surgical clinical practical application to basic pharmacology teaching. This innovation not only verifies the feasibility of 3DIPT technology in cross-disciplinary medical education applications. It also lays a foundation for subsequent extension to complex systems such as cardiovascular and cerebrovascular systems (Bontempo et al., 2024; El Khoudary et al., 2020). The team’s mature 3DIPT technology system can be extended to the teaching of more basic medical disciplines in the future based on existing experience. Combined with the SFPC model, it can provide visualization teaching tools for abstract disciplines such as pathophysiology and immunology. It also provides a new path for the deep integration of “clinical practice-basic teaching-social value” in medical education. This is in line with the development trend of pharmacology teaching toward precision, visualization, and practicality.

### 5.5 Study limitations and prospects

This study has certain limitations. First, the grouping method is non-random, based on interns' commuting and time availability. Although the balance test of baseline data (P > 0.05) reduced the impact of measurable confounding factors, residual confounding and potential selection bias cannot be completely avoided. Future studies can expand the sample size and further optimize the grouping design by combining stratified matching. Second, the observation period is short (6 months). In the future, the follow-up time can be extended to further evaluate the long-term impact of the SFPC+3DIPT teaching model on interns' knowledge retention, professional identity, and career decisions (such as the intention to apply for pharmacology-related majors). Third, this study only focuses on the teaching of urogenital system drugs. In the future, this model can be extended to other systems such as cardiovascular, cerebrovascular, and digestive systems to verify its universality (Bontempo et al., 2024; El Khoudary et al., 2020).

These limitations are fully and transparently reported in this study, and all conclusions are interpreted strictly within the scope of the study design.

It is worth noting that studies on the association between menopausal hormone therapy and cardiovascular diseases have confirmed the multi-system impact of drug therapy in this field (Bontempo et al., 2024). This provides important clinical literature support for our future extension to pharmacological teaching in fields such as coronary artery stenosis and lower extremity vascular lesions. As a preliminary exploration of the SFPC+3DIPT model in pharmacology teaching, this study has certain limitations. However, it provides a practical and feasible new idea for solving the abstract problem of traditional pharmacology teaching. It is in line with the innovative development needs of medical education and is expected to be recognized by editors and reviewers in the field of pharmacology.

In conclusion, this study provides an innovative and efficient solution for pharmacology teaching through the organic combination of 3DIPT physiological and pharmacological visualization and the SFPC collaborative model. This model not only improves interns' mastery of drug knowledge and comprehensive learning abilities, but also provides a new idea for the integration of “theoretical abstraction-practical visualization-social value” in medical education, with good popularization and application prospects.

## 6 Conclusion

The School-Family-Physician Collaborative (SFPC) model combined with 3D Intelligent Printing Teaching (3DIPT) significantly improves the mastery of pharmacology knowledge in undergraduate clinical medicine interns during their internship period. At the same time, the model enhances core competencies of medical professionals, including medical interest, self-directed learning ability, doctor-patient communication ability, and transformative learning ability.

Specifically, the SFPC+3DIPT model aligns with the core tenets of embodied cognition theory, and within the scope of this teaching research topic, it significantly supports learners’ understanding of the underlying physiological and pharmacological principles. It transforms abstract concepts—such as hormonal regulation pathways, anatomical structures, and drug action targets—into visual, embodied learning experiences, as evidenced by statistically significant improvements in pharmacology and pharmacy knowledge directly related to physiological principles, as well as improved comprehension and application of these principles in simulated clinical contexts.

This teaching model effectively meets the demand for visualized and interactive teaching in pharmacology education and addresses the abstract nature of pathophysiological mechanisms in traditional pharmacology teaching. As clinical medical education moves toward competency-based training, SFPC+3DIPT shows great potential to promote the training of high-quality medical talents and improve overall clinical education levels. Future multi-center, longitudinal studies are needed to verify the long-term effects of this assisted teaching model, and further work is required to optimize the model for wider application in basic disciplines such as pharmacology.

## Data Availability

The original contributions presented in the study are included in the article/supplementary material, further inquiries can be directed to the corresponding authors.
